# From non-covalent binding to irreversible DNA lesions: nile blue and nile red as photosensitizing agents

**DOI:** 10.1038/srep28480

**Published:** 2016-06-22

**Authors:** Hugo Gattuso, Vanessa Besancenot, Stéphanie Grandemange, Marco Marazzi, Antonio Monari

**Affiliations:** 1 Université de Lorraine – Nancy, Theory-Modeling-Simulation SRSMC, Vandoeuvre-lès-Nancy, France; 2 CNRS, Theory-Modeling-Simulation SRSMC, Vandoeuvre-lès-Nancy, France; 3 Université de Lorraine – Nancy Santé, Biologie, Signal - CRAN, Vandoeuvre-lès-Nancy, France; 4 CNRS, Santé, Biologie, Signal, CRAN, Vandoeuvre-lès-Nancy, France

## Abstract

We report a molecular modeling study, coupled with spectroscopy experiments, on the behavior of two well known organic dyes, nile blue and nile red, when interacting with B-DNA. In particular, we evidence the presence of two competitive binding modes, for both drugs. However their subsequent photophysical behavior is different and only nile blue is able to induce DNA photosensitization via an electron transfer mechanism. Most notably, even in the case of nile blue, its sensitization capabilities strongly depend on the environment resulting in a single active binding mode: the minor groove. Fluorescence spectroscopy confirms the presence of competitive interaction modes for both sensitizers, while the sensitization via electron transfer, is possible only in the case of nile blue.

DNA photosensitization^1^,^2^ is a fundamental phenomenon with implication to public health, medicine and pharmacology. Indeed DNA is constantly exposed to different, endogenous and exogenous, sources of stress that may result in DNA bases or backbone damages^3^,^4^,^5^. The sources of stress include free radical or reactive oxygen species, as well as radiation and exposure to UV-visible light. DNA lesions may be recognized and efficiently repaired by the activation of specifically tailored enzymes, however if the cycle of damages and repair is unbalanced one may assist to an accumulation of lesions. These facts could induce cellular apoptosis or, on the other hand, produce mismatches in the replication cycles that may ultimately result in mutations and carcinogenesis^6^,^7^. Concerning UV-visible induced damages one should divide between direct damages, resulting from UVB radiation absorption by DNA bases, or indirect damages caused by external agents^3^. The former have been the subject of intense theoretical and experimental studies and mostly result in bases dimerizations (such as cyclobutane pyrimidine dimers or the 6-4PP photodamage)^8^,^9^,^10^,^11^,^12^,^13^,^14^,^15^,^16^. On the other hand the interaction between DNA and external chromophores may lead to DNA damages subsequently to the exposure to UVA or even visible light^2^,^17^. The former process is known under the name of photosensitization and requires the establishment of long-lived DNA/drug aggregates, the absorption of light by the drug and its subsequent photophysical or photochemical evolution leading to the lesion^18^. In many cases DNA photosensitization proceeds following an intersystem crossing that promotes the population of the sensitizer triplet manifold^19^. This can subsequently evolve via triplet-triplet energy transfer, usually toward thymine nucleobases^17^,^20^, or via the activation of singlet oxygen (type II photosensitization), the latter being known to selectively react with guanine^21^,^22^. Other photochemical channels leading both to bases and backbone lesions or strand breaks have been reported, such as hydrogen abstraction^23^,^24^,^25^. Finally one should recognize the possibility of photo-induced electron transfer from the DNA bases to the photosensitizer, happening both from triplet or singlet states, in the so-called type I photosensitization^4^,^26^.

DNA photosensitization can be seen as an environmental threat since many common pollutants such as benzophenone and acetophenone are known to produce DNA photosensitization following complex and varied mechanisms^17^,^27^,^28^. As such, those molecules greatly enhance the dangerous spectral width capable of inducing potentially harmful DNA lesions.

On the other hand photosensitization can also be exploited for medical and pharmacological reasons. Indeed, photosensitization can be seen as the base of the photodynamic therapy commonly used against skin pathologies and some types of cancers^29^,^30^,^31^,^32^. This technique, already used under the name of heliotherapy in ancient Egypt^33^, consists in administering the patient with a DNA photosensitizer followed by the selective irradiation of the area of the lesion, in order to selectively induce apoptosis of the ill cells without affecting general methabolic pathways and hence reducing side effects^34^.

Photodynamic therapy is usually performed via type II photosensitization, i.e. inducing singlet oxygen production. However, this aspect limits its application against solid tumors, which are known to exhibit hypoxia conditions^35^. On the same spirit it is strongly favorable to consider red, or infrared, absorbing photosensitizers since longer wavelength photons penetrate much deeper into tissues than shorter wavelength ones^26^. As a matter of fact the development of two–photons absorption photosensitizers has shown a considerable growth in the last years^36^,^37^.

The use of long wavelength absorbers capable to perform type I photosensitization (electron transfer) could be seen as a way to tackle both hypoxic conditions and the need of relatively penetrating radiations^38^.

Nile blue (NB) and nile red (NR) as sketched in Fig. 1 are two fluorescent dyes having a rather wide use in different areas and most notably in cellular biology^39^,^40^. Indeed, and especially NR shows an environment dependent fluorescence that is switched on only in non-protic media such as lipid bilayers and membranes. Recently, we have elucidated the optical properties of the two dyes in water environment, most notably showing the importance of the inclusion of dynamic and vibrational effects in the calculation of the absorption spectrum, including the global capability of Time Dependent Density Functional Theory (TD-DFT) to correctly reproduce higher level and experimental results^41^.

NB, cationic, is characterized by an intense band at around 600 nm that is responsible for its intense blue color that gives its name. On the other hand, the neutral NR absorb at shorter wavelengths. Although both molecules could experience intramolecular charge-transfer, the analysis of the electronic density rearrangement upon excitation has clearly revealed that the lowest transition is local and of π–π* nature^41^.

In a recent work by Hirakawa *et al*.^42^ it has been observed that NB is able to induce DNA lesions under the effect of visible light. The authors have postulated that the photosensitization is initiated by a photo-induced electron transfer from NB to a nearby guanine. The authors also hypothesize that the mechanism’s efficiency is enhanced by a second backward charge transfer that will reinstate the original NB form, while the produced guanidinium cation will further evolve to produce DNA lesions.

In the present contribution we want to investigate the interaction between NB, NR, and B-DNA, in order to unravel, using different modelling techniques, the photochemical pathways opened by this interaction. The results of simulations will also be compared with experimental spectroscopic (absorption and fluorescence) analysis. Our characterization of the charge transfer properties and phenomena at a molecular and electronic level will allow us to discriminate between the sensitizing capacity of NR and NB. Furthermore, since it has been shown that optical properties are strongly environment dependent^41^, we will be able to assess the role of the non-homogeneous molecular environment on the photochemistry of the two dyes. In particular the existence of preferential interaction modes with DNA, leading to easier sensitization pathways will be taken into account. This will be accomplished with a full multiscale protocol combining the use of state-of-the-art molecular dynamics with hybrid quantum mechanics/molecular mechanics (QM/MM) methods^43^. Since we are mostly interested in charge transfer phenomena, and in order to limit the complexity of the problem, we will consider only oligonucleotides constituted by guanine-cytosine sequences. Indeed, due to the difference in redox potential between the DNA nucleobases, it is established that only guanine may reduce the great majority of type I sensitizers^44^,^45^.

Furthermore, in order to assess sequence selectivity of photosensitization we performed absorption and fluorescence spectroscopic tests on both guanine-cytosine and adenine-thymine DNA double-strands.

## Results

### Interaction with DNA

**Figure 2:**
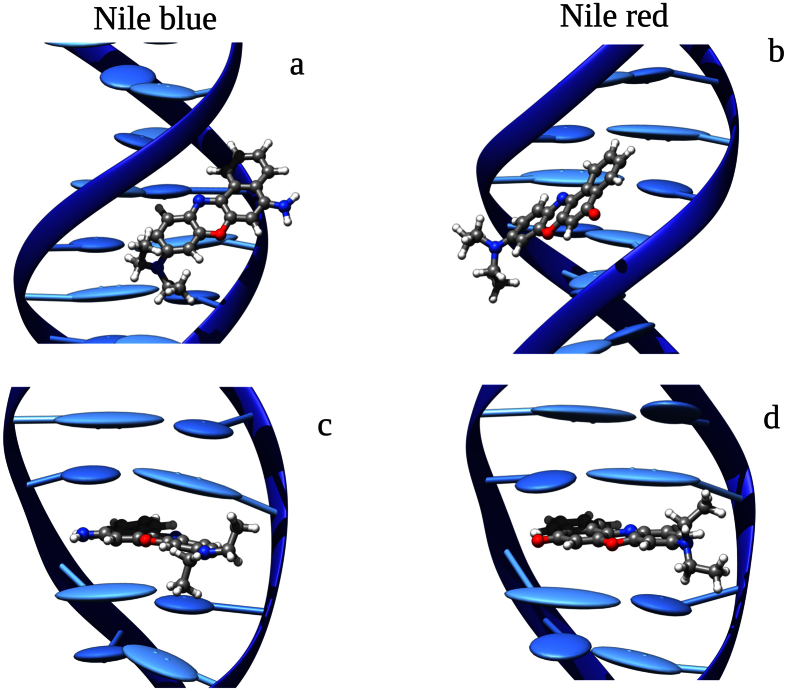
DNA binding interaction modes: selected MD snapshots of NB in minor groove binding (**a**) and intercalated (**c**). NR in minor groove binding (**b**) and intercalated (**d**).

The first step in assessing the potentially different photochemistry of DNA sensitizers is to unravel its or their binding modes with the biological system. From the MD simulation it turned out that both NR and NB present two stable interaction modes with the DNA double strand (Fig. 2). Indeed, in both cases one can observe the presence of minor groove binding and intercalation. The positive charge held by NB can certainly justify minor groove binding due to electrostatic interactions with the charged phosphate groups in the backbone. In the case of NR minor groove binding is mostly driven by a combination of dispersion and labile hydrogen bonds interactions. Intercalation, on the contrary, is largely driven by dispersion interactions and the π-stacking between sensitizers and the nucleobases in the intercalation pocket. Even if costly calculations of binding free energies would be necessary to ultimately assess the relative stability of the modes, one can presume that NB would probably favor minor groove binding instead of intercalation. In this latter case NB’s positive charge will be surrounded by the hydrophobic environment of the DNA core; for the opposite reasons we could probably state that NR will be more prone towards intercalation.

The structural deformations induced on DNA by the interactions with the sensitizers are quite standard for those kind of DNA non-covalent binding modes and almost indistinguishable between the two sensitizers.

**Figure 3:**
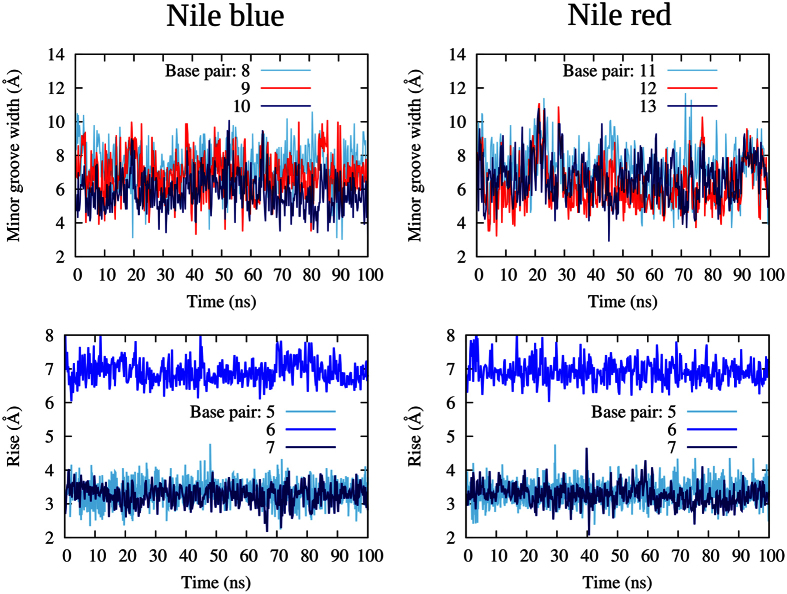
DNA structural analysis: time series for the minor groove width for NB and NR in minor groove binding and rise parameter for NB and NR in intercalation. The chosen base pairs are the ones interested by the interaction with the sensitizers together with one non-interacting pair as reference.

Almost no peculiar deformation in the minor groove parameters can be evidenced for the minor groove binding (Fig. 3 top), while in the case of intercalation the distance between the bases (rise parameter in Fig. 3) is doubled for the bases in between which NB or NR slips. This feature is quite common in the case of intercalation and allows the creation of the intercalation pocket able to accommodate the sensitizer. The other structural parameters are much less affected, and most notably the bending of the axis stays close to its ideal value all along the trajectory, indicating a global stability (see Supplementary Information).

### Absorption spectra

**Figure 4:**
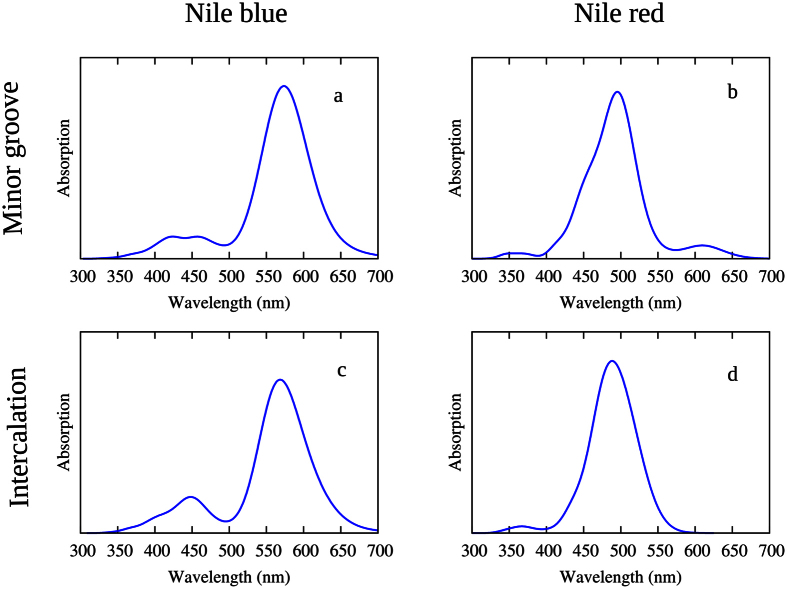
Simulated absorption spectra of NB and NR interacting with B-DNA. NB in minor groove binding (**a**) and intercalated (**c**). NR in minor groove binding (**b**) and intercalated (**d**).

The calculated absorption spectra for NB and NR in interaction with DNA (minor groove binding and intercalation mode) are reported in Fig. 4. The main optical characteristic of the two dyes are quite well reproduced, in particular the low energy intense peak corresponding to the S0 → S1 transition that will dominate the subsequent photophysics.

It is also noteworthy to see that the interaction with DNA has only a very slight effect on the position of the absorption peaks. Indeed, both interaction modes give, for the same sensitizers, almost the same excitation energy that also happens to be quite close to the one obtained in aqueous environment^41^, indicating a quantitative agreement between implicit and explicit water solvent models.

**Figure 5:**
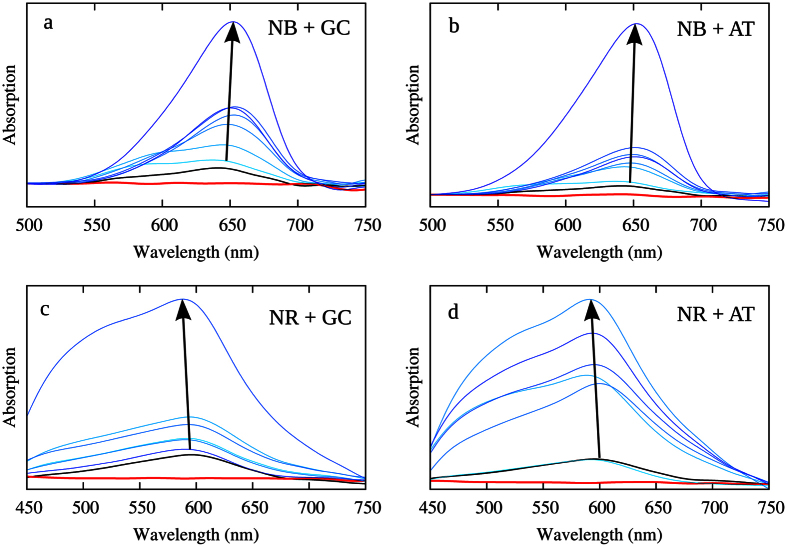
Experimental absorption spectra of NB (**a**,**b**) and NR (**c**,**d**) interacting with polydA-dT and polydG-dC DNA strands. Black and red curves represent the absorption of solvated sensitizer (NB and NR) and DNA, respectively; blue curves represents different DNA concentrations: from the lowest, represented by light blue curves, to the highest shown in dark blue (see Methods). The sensitizer concentration is kept constant.

Finally, and to prove the interaction with DNA, we report the absorption spectra for NB and NR obtained with different concentrations of DNA (Fig. 5). The change of the intensity of the NB (NR) characteristic band with the increasing concentration of DNA is clearly indicative of the development of non-covalent interactions and hence of the fact that both dyes are able to give raise to stable non-covalent aggregates with DNA. On the other hand, the negligible change in the position of the maximum also confirms our theoretical results. The consistent red-shift compared to our theoretical results can be adequately recovered by introducing ab-initio electronic dynamic correlation, as already evidenced in our previous study on both organic dyes^41^. Interestingly, it also evident that NB absorption maximum falls in the phototherapeutic window (620–850 nm), while both NB and NR long-wavelength tails significantly overlap with the phototherapy window.

### Charge transfer mechanisms

**Figure 6:**
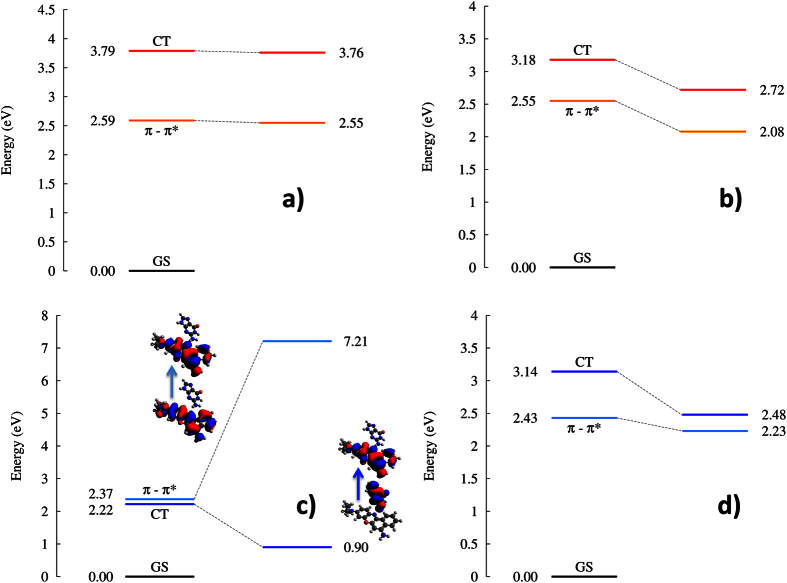
Jablonski (energy) diagram, showing the evolution of locally excited (π–π*)and charge transfer states (CT), from the Franck–Condon region to the respective excited state minima. (**a**) NR in minor groove binding, (**b**) NR in intercalation, (**c**) NB in minor groove, (**d**) NB in intercalation. Energies for the optimized structures are reported with reference to the energy of the ground state at the lowest excited state minimum. NTOs for NB in minor groove are given in panel (**c**) (see Supplementary Information for the other cases).

In order to assess the photoinduced charge transfer mechanism by NB and NR we have performed simulations at the QM/MM level, including in the QM partition the sensitizer and one nearby guanine. Subsequently the geometry of the lowest excited states has been optimized in order to obtain the corresponding Jablonski diagram and hence to infer the photochemical pathways (Fig. 6). In particular, and already at the Franck–Condon region, in addition to the local transitions we evidence the presence of a long-range charge transfer state. Its analysis, in terms of natural transition orbitals (NTO) and ϕ_S_ index^46^ (see Supplementary Information for details), confirms that the state correspond to an electron transfer from the guanine to the sensitizer.

In the case of NR the charge transfer state is quite high in energy: at Franck-Condon the charge transfer state is 0.65 eV (intercalation) and 0.86 eV (minor groove binding) higher than the lowest-energy local excited state. Even upon optimization the charge transfer state always lays higher in energy than the local π–π* state and their relative energy only changes negligibly for both interaction modes. As a consequence we may conclude that the sensitization through electron transfer is precluded for NR, since the population of the charge transfer state is most unlikely to happen for energetic reasons.

On the contrary NB shows a much interesting pattern. Indeed, in the case of intercalation the charge transfer state at Frank-Condon is higher in energy (0.71 eV) and upon optimization it does not cross the lowest-energy local excited state, however the energy difference is strongly reduced and the states become quasi-degenerate laying only 0.25 eV far apart. Even more appealing, the lowest-energy singlet excited state for minor groove binding is the charge-transfer, lying 0.15 eV lower than the local state at Frank-Condon. This energetic order is preserved, and even amplified, upon optimization of both local and charge-transfer states. The local π–π* excited state is now destabilized while the charge transfer one is strongly stabilized ultimately leading to an energy difference of about 6.0 eV. This situation could seem quite unlikely since one could expect that the π-stacking between NB and guanine, characteristic of intercalation, should favor the coupling of the two chromophores and hence the charge transfer. However, it has to be recalled that in the case of intercalation charge separation should take place in a strongly hydrophobic environment, hence being less favorable than the one happening in the hydrophilic and charged minor groove region.

Our results clearly point toward the fact that charge transfer from guanine to NB is feasible, but is environmentally controlled: it can take place only in the case of minor groove binding. Since the local π–π* state is always much optically brighter than the charge transfer, one may expect the photophysics to proceed in the following way: light absorption will induce the transition to the local π–π* state (S_2_ for NB in minor groove binding), subsequently the system will go through an internal conversion that is expected to be fast due to the low energy difference and the short distance between the chromophores, leading to the population of the charge transfer state (S_1_). This will hence promote the charge separation with the formal production of a guanidinium cation that will subsequently evolve to induce further DNA lesions.

### Fluorescence spectra

**Figure 7:**
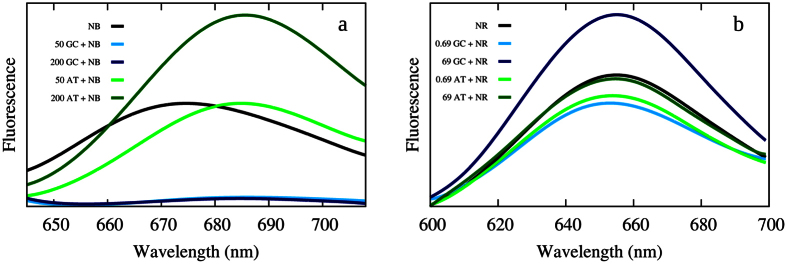
NB (**a**) and NR (**b**) experimental fluorescence spectra. Black curve refers to the fluorescence of the solvated NB (10 μM) or NR (690 μM). Green curves and blue curves refers to the fluorescence of the sensitizer in presence of polydG-dC or polydA-dT double strand, respectively. DNA concentrations are given in ng/L and the concentration of the sensitizer is kept constant.

The presence of electron transfer in the case of NB can also be confirmed by performing experimental fluorescence spectra in presence of polydA-dT or polydG-dC DNA duplexes. Indeed, since electron transfer should be possible only from guanine, one can expect a strong fluorescence quenching in the case of guanine rich strands, while the polydA-dT strand should have a differential effect. This can be seen in Fig. 7a): it is evident that the fluorescence of NB in presence of polydG-dC is almost entirely quenched, hence confirming the presence of an accessible charge transfer state that strongly limits the life-time of the potentially emissive local π–π* state.

**Figure 8:**
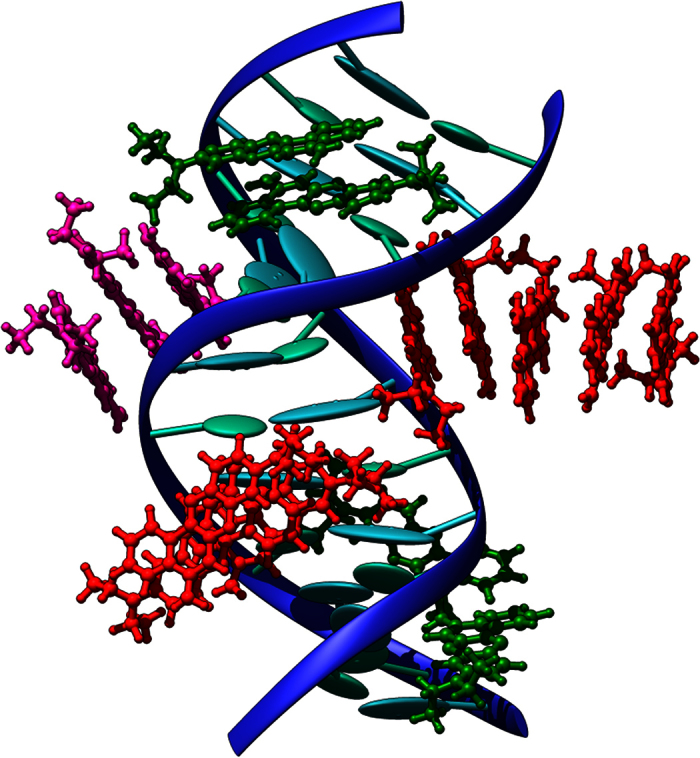
Representative snapshot of a DNA double strand in presence of an excess concentration of NB. Note the spontaneous formation of minor-groove binding as well as of π-stacked aggregates. The different interaction conformations are represented with different color of NB.

However, as also reported in the Supplementary Information, the fluorescence behavior of NB interacting with polydA-dT is much more complex. Indeed after a quenching happening at small concentrations one witnesses a sharp enhancement of the emission intensity and hence of the quantum yield, that becomes even higher than the one of solvated NB alone. This fact is usually interpreted with the presence of two interaction modes^47^. The quenching at low concentration is usually seen as the mark of electrostatic (groove binding) interaction modes that at higher concentration are in competition with intercalation. This aspect is also confirmed by Fig. 8 showing the MD simulation of a solution of DNA double strand in which an excess of NB is present. The spontaneous formation of stable aggregates and clusters of NB in the minor groove after 50 ns is clearly evidenced by a representative snapshot of the 100 ns dynamics.

Those π-stacked structures may be regarded as the ones responsible for the initial quenching of the fluorescence, in accordance with the observed loss of emission intensity in the case of stacked organic fluorophores. Intercalation that was not spontaneously observed probably due to a higher kinetic barrier could, most probably, become competitive at higher concentration and be responsible of the subsequent fluorescence enhancement. On the other hand, as reported in Fig. 7b, NR fluorescence is not quenched by the polydG-dC strand, and both DNA sequences show the characteristic fluorescence enhancement at high concentrations. Globally it is evident that the fluorescence measurement strongly confirms our prevision of the presence of two competitive binding modes, as well as the photoinduction of electron transfer through guanine by NB correlated to the almost total fluorescence quenching.

## Discussion

With a combined use of molecular modeling and experimental spectroscopy we have unraveled the different interactions modes, and photochemical pathways of two organic dyes (NB and NR) interacting with B-DNA. In particular we have evidenced the presence for both drugs of two competitive interaction modes, namely intercalation and groove binding. The subsequent QM/MM exploration of the different excited states has shown that while NR is unable to induce any sensitization of the DNA through electron transfer, NB may indeed give rise to a net transfer of an electron from DNA to the dye. However, the sensitization properties of NB are strongly dependent on its molecular environment and in particular on its binding mode. Indeed, while in intercalation, probably because of the embedding in a hydrophobic environment, charge separation is unfavorable. On the contrary, a highly feasible and energetically favored pathway for electron transfer is evidenced in the case of groove binding. This suggests that NB, also due to its absorption in the red part of the spectrum also overlapping the phototherapeutic window, could be used as a sensitizer agent in hypoxy phototherapy to induce DNA lesions via an electron transfer mechanism (Type I).

Absorption and fluorescence spectra have confirmed the presence of two competitive interaction modes. Furthermore, the almost complete quenching of fluorescence by guanine rich sequences, compared to the more complex behavior in the case of polydA-dT, is strongly indicative of a charge transfer sensitization. In the following we plan to completely characterize the time-evolution of the different involved excited state by using non-adiabatic molecular dynamics, and hence have access to characteristic times connected with the sensitization process. Unraveling at electronic and molecular level the mechanism hidden behind sensitization, and also its dependence on the environment, will ultimately greatly help the rational design of novel (photo)-therapeutic agents interacting with DNA.

## Methods

All simulations were performed on a TIP3P^48^ water solvated polydG-dC hexadecamer, including counterions. The stability of each DNA-photosensitizer complex was validated after a pre-equilibration step and a final 100 ns MD simulation production. The amber99 force field parameters^49^ with bsc0 correction^50^ were applied to the DNA strand, while GAFF parameters^51^ coupled to RESP fitted atomic charges were considered for NB and NR. The MD simulations were carried out using the GPU CUDA version of amber15^52^, combined with Curves+ as a tool to analyze DNA structural distortions caused by the sensitizers^53^.

Hybrid quantum mechanics/molecular mechanics (QM/MM) simulations were performed to correctly model electronic excited states. The QM region comprises the sensitizer (NB or NR) and the nearest guanine nucleobase. The time dependent – density functional theory was used in the framework of a TD-DFT/Tinker protocol for excited state optimization, based on electrostatic embedding and hydrogen link atom schemes. More in detail, the hybrid meta exchange-correlation M06-2X functional^54^ was selected, with a 6−31+G(d) basis set, as it can properly describe charge transfer mechanisms. Moreover, it was shown to be in agreement with ab initio multiconfigurational CASPT2 calculations on NB and NR^41^. All QM/MM calculations were performed with a locally modified^43^,^55^ version of the Gaussian09 code^56^, coupled to Tinker^57^.

The fluorescent and absorption spectra have been acquired using a fluorescent microplate reader Xenius SAFAS and a Nanodrop system from Thermo Scientific, respectively. Spectra have been registered with increasing concentration of polydA-dT and polydG-dC probes (Sigma) for a fixed amount of NB and NR. For NB assays, a range of polydA-dT and polydG-dC from 1 to 200 ng/μL was used and added to a 10 μM of NB (solubilized in water) solution (emission spectra were acquired with an excitation wavelength at 625 nm). For NR assays, a range of polydA-dT and polydG-dC from 0.69 to 69 ng/μL was used and added to a 690 μM of NR (solubilized in methanol) solution (emission spectra were acquired with an excitation wavelength at 560 nm).

## Additional Information

**How to cite this article**: Gattuso, H. *et al*. From non-covalent binding to irreversible DNA lesions: nile blue and nile red as photosensitizing agents. *Sci. Rep.* **6**, 28480; doi: 10.1038/srep28480 (2016).

## Supplementary Material

Supplementary Information

## Figures and Tables

**Figure 1:**
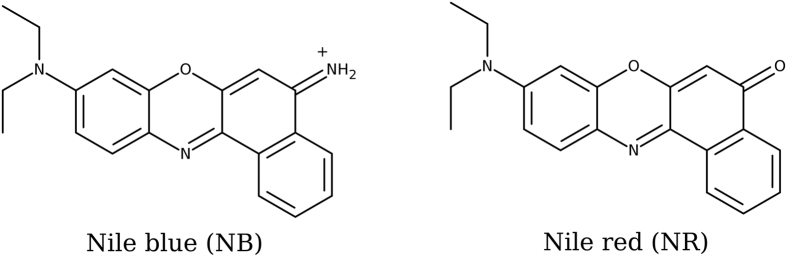
Nile blue and nile red molecular formulae.
