# Supplementary material for: From non-covalent binding to irreversible DNA lesions: nile blue and nile red as photosensitizing agents
Source: Sci Rep. 2016 Jun 22;6:28480. doi: 10.1038/srep28480 (PMC4916457; doi:10.1038/srep28480)
Supplement: Supplementary Information [file srep28480-s1.pdf]

Supplementary Information

From non-covalent binding to irreversible DNA lesions: nile blue and nile red as photosensitizing agents

Hugo Gattuso,[a,b] Vanessa Besancenot,[c,d] Stéphanie Grandemange,[c,d] Marco Marazzi,[a,b]* and Antonio Monari[a,b]*

[a)] Université de Lorraine – Nancy, Theory-Modeling-Simulation SRSMC, Vandoeuvre-les-Nancy, France
[b)] CNRS, Theory-Modeling-Simulation SRSMC, Vandoeuvre-les-Nancy, France
[c)] Université de Lorraine – Nancy Santé, Biologie, Signal - CRAN, Vandoeuvre-les-Nancy, France
[d)] CNRS, Santé, Biologie, Signal, CRAN, Vandoeuvre-les-Nancy, France

Content

1) Computational details

2) DNA structural analysis

3) Molecular orbitals

4) Experimental fluorescence spectra

5) MD simulation of DNA with NB in excess concentration

1) Computational details

DNA-NB/NR complexes were placed in a 75×80×100 Å rectangular parallelepiped box, filled with 16830 TIP3P water molecules. To simulate the bulk phase, periodic boundary conditions were applied, including a cutoff to turn off the interactions between atoms that are more than 9.0 Å apart. The system was neutralized with 29 (30) Na^+ cations in the case of NB (NR) complexes.

When generating the force field parameters for NB and NR, the atomic charges were calculated by the RESP fitting protocol using Hartree–Fock quantum calculations and the 6-31G(d) basis set, as a commonly applied for organic molecules.

All generated initial conditions (*i.e.* intercalation, insertion, minor groove and major groove binding modes) were pre-equilibrated as follows: an energy minimization of 8000 steps was performed (4000 steps using the steepest descent algorithm followed by 4000 steps using the conjugate gradient algorithm) prior to (*i*) 200 ps molecular dynamics (MD) to heat the system up to 300 K in the *NVT* canonical ensemble, followed by (*ii*) a 200 ps MD equilibration in the NPT isothermal-isobaric ensemble at 300 K and 1 atm to acquire the correct density.

Finally, a 100 ns MD production run was performed for each DNA-NB/NR binding mode, so to validate the complex stability, and therefore select intercalation and minor groove binding as the only stable modes for both DNA-NB and DNA-NR complexes.

During all pre-equilibration and production MD simulations, the shake algorithm was applied to chemical bonds involving hydrogen atoms. Besides, the temperature was kept fixed at 300 K by using Langevin dynamics, while the pressure was maintained at 1 atm by the Monte Carlo thermostat.

The applied MD simulation protocol was also validated analyzing the behavior of the DNA model non interacting with the sensitizer and showing that the main structural and dynamic features of the B form were recovered.

For setting up QM/MM calculations, one hydrogen link atom was placed at the border between the selected guanine base pair and the sugar moiety, more in detail between N9 of the guanine and C1' of the sugar. The whole MM region (the rest of the DNA double strand, all water molecules and counterions) was allowed to minimize its energy at each optimization step.

The simulated absorption spectra (Figure 3 of the main text) were built by considering the energy difference and oscillator strength between the electronic ground state and the ten lowest excited singlet states (at the TD-DFT/amber99 level of theory) of 100 randomly selected structures along the MD trajectory. A final convolution using Gaussian functions with full-width at half-length of 0.2 eV was performed to simulate spectral band shape.

2) DNA structural analysis

During the MD simulations, an analysis of the DNA geometrical parameters was performed with the Curves+ program. Figure S1 shows the parameters mainly involved in the description of the intercalation and minor groove binding modes.

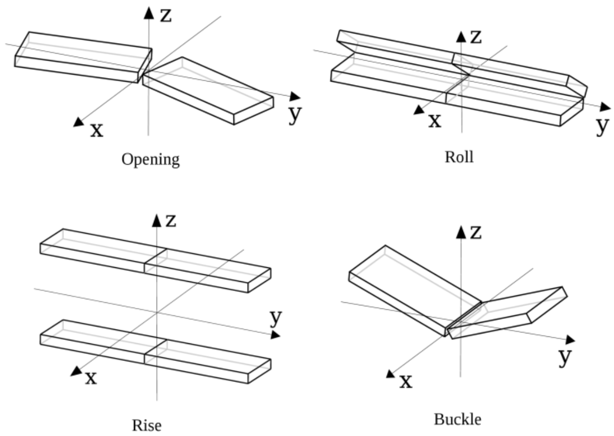

Figure S1. Pictorial description of the rigid body parameters used to describe the geometry of the DNA base pairs.

Figures S2 and S3 show the main geometrical parameters involved in the minor groove binding and intercalation modes, respectively. As can be seen, the intra base parameter (left column) always indicates that the DNA stays as a double strand. The rise parameter properly shows that the distance between two subsequent base pairs is relevant only for the intercalation mode (Figure S3, middle column) between base pairs 5 and 7; while the groove parameter indicates that the DNA-sensitizer complex formation does not affect the minor groove geometry (Figure S2). The distortion appearing in the DNA-NR intercalated complex between the 10th and the 12th base pair (Figure S3 top) are due to a local fluctuation of a DNA strand end, in any case not affecting the analysis of the intercalated mode located between the 5th and the 7th base pair (Figure S4).

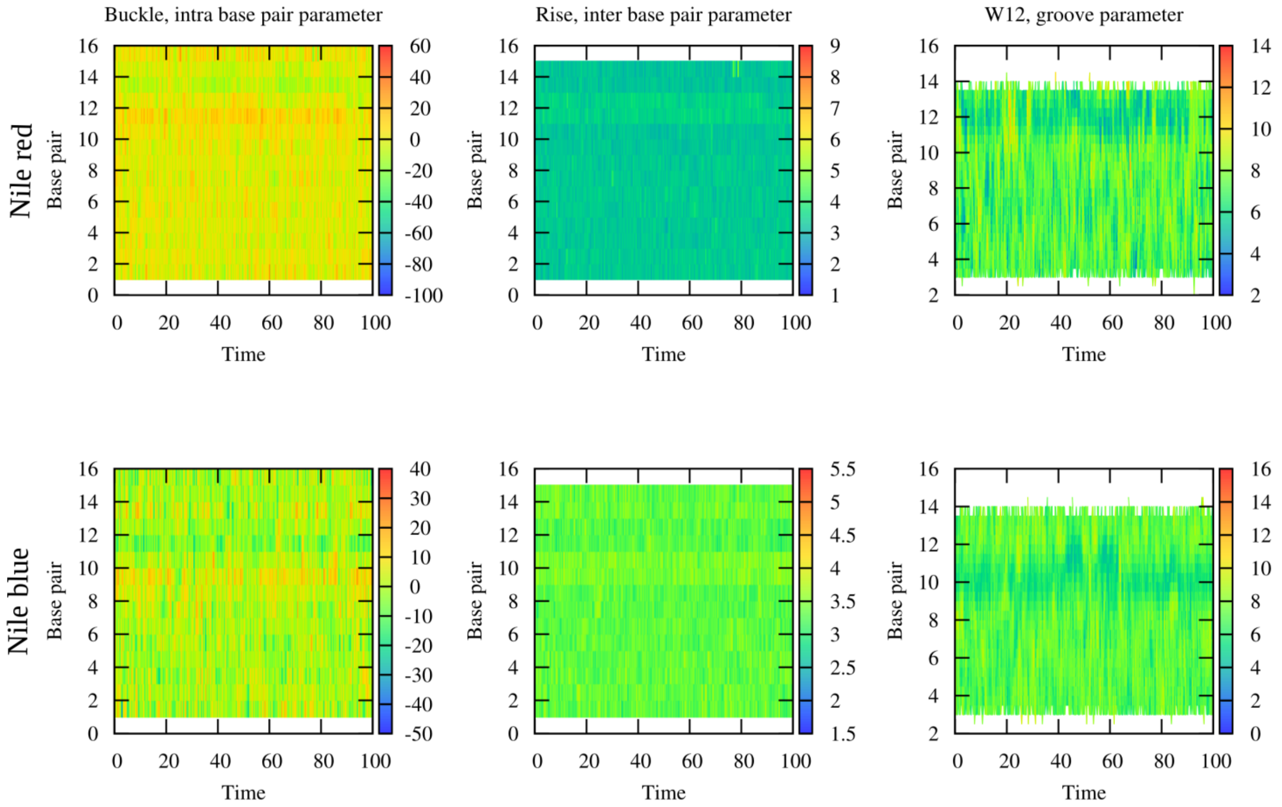

Figure S2. DNA-NR (top) and DNA-NB (bottom) main geometrical parameters involved in the minor groove binding mode.

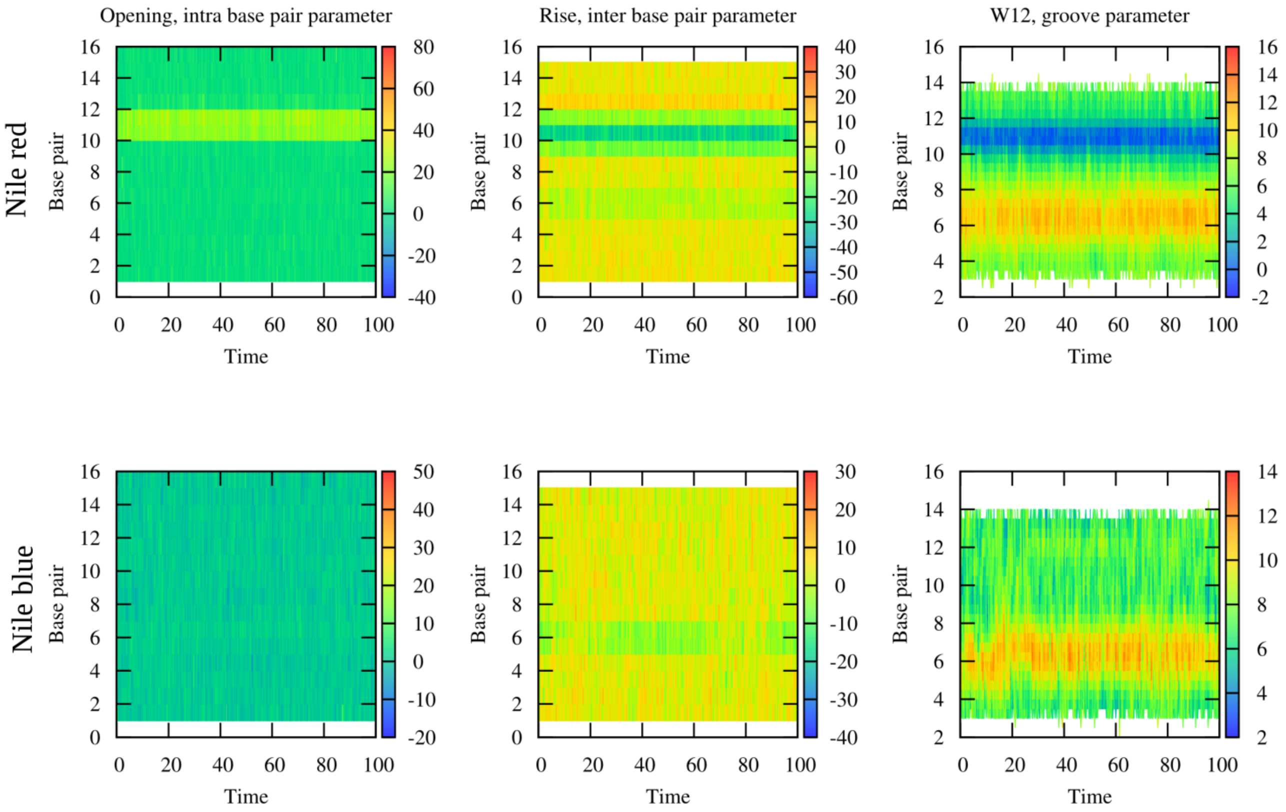

Figure S3. DNA-NR (top) and DNA-NB (bottom) main geometrical parameters involved in the intercalation mode.

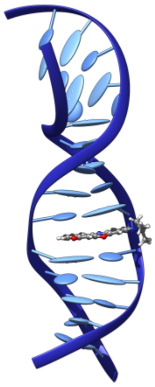

Figure S4. Representative snapshot of the DNA-NR complex in intercalation.

3) Molecular orbitals

The natural transition orbitals involved in the description of the electronic transitions to the locally excited and charge transfer excited state are shown in Figures S5 (for NB) and S6 (for NR). For the reader convenience we remind that NTO analysis is based in a singular value decomposition of the transition density matrix that allows to represent the density rearrangement upon excitation using only one orbital couple. The occupied orbital represent the regions from were electronic density have been depleted (i.e. a sort of hole density), while virtual orbital refers to the regions were electrons are accumulated. The nature of the excited states were also assessed using the ϕ_S index, that assumes values close to 1.0 for local states and close to 0.0 for charge transfer ones. In our case the local states had values close to 0.8 while charge-separated one assumed values of around 0.4. NTOs and ϕ_S have been calculated using the locally developed Nancy_Ex code freely available on the net under GPU license (https://sourceforge.net/projects/nancyex/)

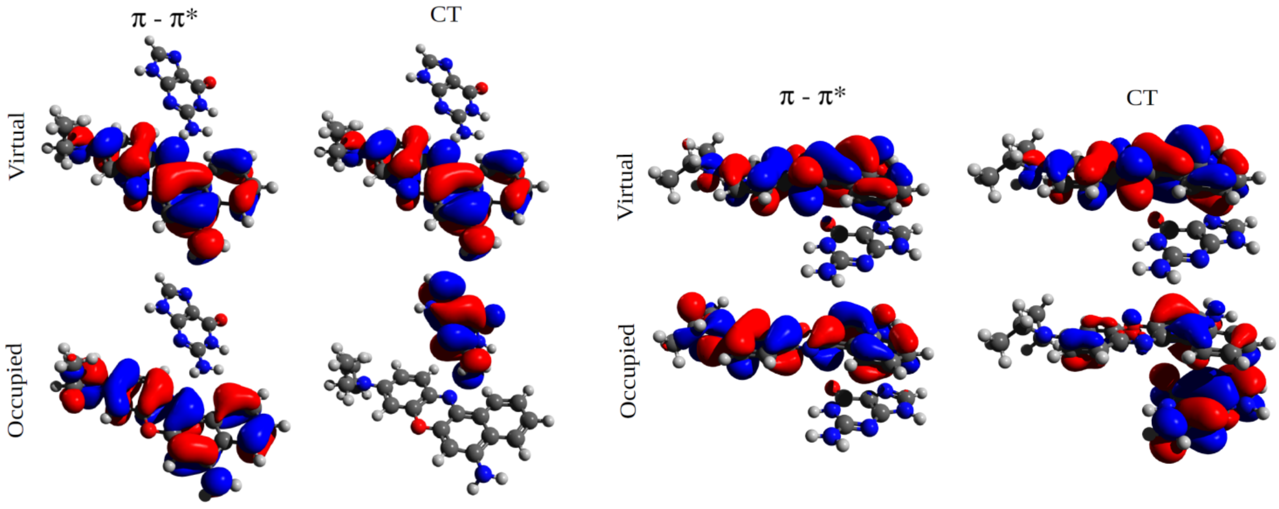

Figure S5. Occupied and virtual natural transition orbitals involved in the electronic transition to the lowest-energy local excited state (π–π*) and to the charge transfer state (CT) of the DNA-NB complex in minor groove binding (left) and intercalation (right).

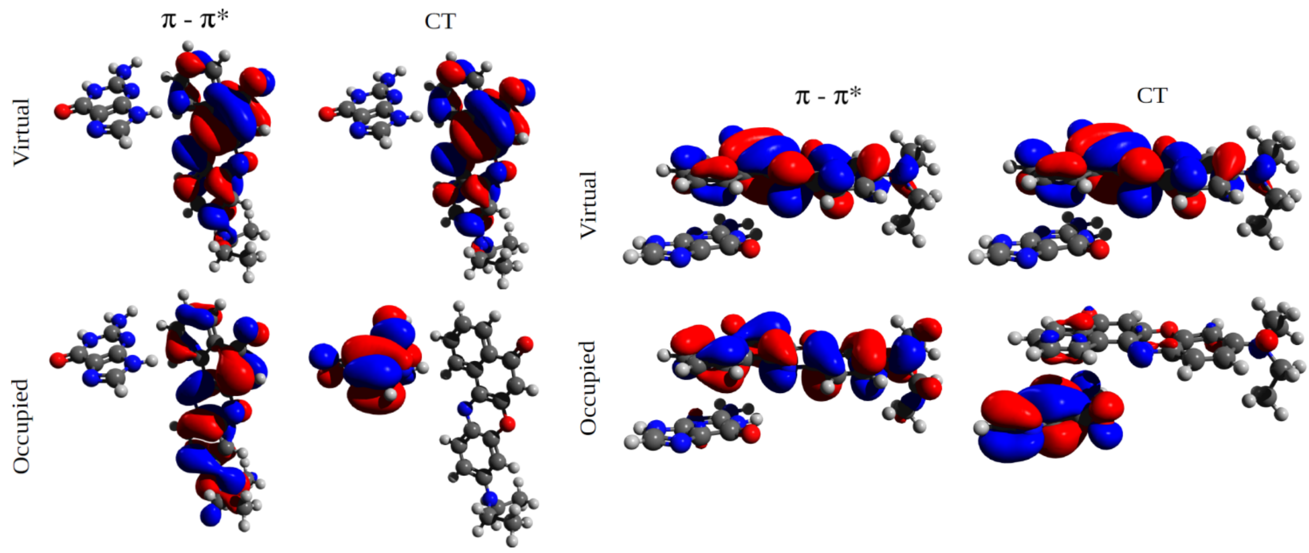

Figure S6. Occupied and virtual natural transition orbitals involved in the electronic transition to the lowest-energy local excited state (π–π*) and to the charge transfer state (CT) of the DNA-NR complex in minor groove binding (left) and intercalation (right).

4) Experimental fluorescence spectra

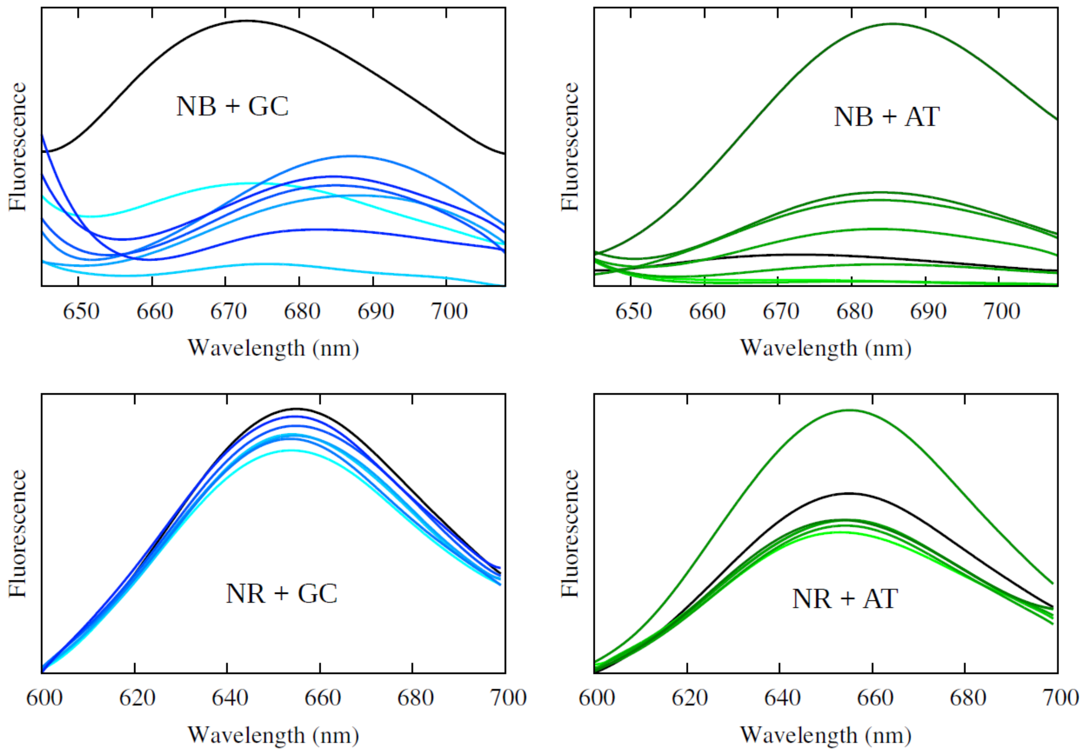

Figure S7. Experimental fluorescence spectra of NB and NR in solution with poly(dG-dC) and poly(dA-dT) (GC and AT, respectively). All concentrations listed in the Methods section of the main text are shown, from the lowest (light blue for GC; light green for AT) to the highest one (dark blue for GC; dark green for AT).

5) MD simulation of DNA with NB in excess concentration

A 100 ns MD production run of poly(dG-dC) in presence of NB in excess concentration (1.25 molecules/base pair) was performed. The position of each NB molecule was chosen randomly.
